# Glucosamine derived hydrothermal carbon electrodes for aqueous electrolyte energy storage systems

**DOI:** 10.3906/kim-2105-35

**Published:** 2021-08-04

**Authors:** Burcu ÜNAL, Rezan DEMİR ÇAKAN

**Affiliations:** Department of Chemical Engineering, Gebze Technical University, Kocaeli, Turkey

**Keywords:** Nitrogen-doped carbon, hydrothermal carbonization, aqueous electrolyte batteries, sodium-ion energy storage

## Abstract

Nitrogen-doped porous hard carbons are synthesized by hydrothermal carbonization method (HTC) using glucosamine as biosource and treated at different carbonization temperatures in nitrogen environment (500, 750, 1000 °C). The electrochemical performances of hard carbons electrode materials for aqueous electrolyte sodium ion batteries are examined to observe the effect of two different voltage ranges (−0.8–0.0) V and (0.0–0.8) V in 1.0 M Na_2_SO_4_ aqueous electrolyte. The best electrochemical performances are acquired for the 1000 °C treated glucosamine (GA-1000) porous carbon sample that provides ~96 F/g capacitance value in the negative voltage range (between −0.8 and 0.0) V. The sodium diffusion coefficient of the GA-1000 carbon calculated by electrochemical impedance measurements is found to be 1.5 × 10^−14^ cm^2^/s.

## 1. Introduction

Major parts of the world’s commercial energy production are obtained from nonrenewable sources such as coal, oil, and natural gas. On the other hand, the damages of the fuels obtained from these sources to the ecosystem are increasing day by day, and the fossil resources are faced to be depleted, thus, searches for new alternative renewable energy sources are accelerating. In order to use renewable power sources effectively, it is necessary to develop more reliable and environmental-friendly energy storage technologies. Existing technologies have some challenging issues in large-scale applications due to the use of flammable electrolyte and high costs electrode materials [1,2]. For instance, the future projections highlight the demand of lithium that will increase by 485 % in 50-years [3], thus, the lack of reserves of lithium resources to meet this need have let to new search directions [4,5]. Having similar physical and chemical properties compared to lithium, sodium-ion based energy storage systems can be preferred as new generation and inexpensive options [6–8]. In addition to that, sodium is abundant in nature, which can be obtained from minerals and salts at a lower cost in comparison with the lithium counterparts.

Regarding electrode materials, carbon materials are often used as anode either in battery or supercapacitor technology due to their high surface area, electrical conductivity, and stability throughout the cycles [9–12]. Generally carbon synthesis methods, which requires several steps such as electric arc discharge techniques, chemical vapor deposition, pyrolysis of organic compounds are used [13]. As an alternative, the hydrothermal carbonization (HTC) has been introduced at this study as a cost-effective and safe method that uses biomass to convert into carbonaceous materials at one step in aqueous medium [14,15]. HTC method is the technique of synthesizing carbonaceous materials under low temperature (<200 °C) and pressure synthesis conditions of carbohydrate solutions with pure water in autoclaves [16,17]. For instance, Sevilla et al. studied the hydrothermal carbonization method of cellulose precursor in which they clarified the reaction mechanisms as hydrolysis of cellulose, the formation of furfural, and the subsequent aromatization and tautomerism steps [18]. Zhao et al. obtained nitrogen-rich hard carbons via HTC method from D(+)-glucosamine. The N-doped carbons derived from nitrogen containing polysaccharides have an increasing effect on the performance of battery and supercapacitor applications. The inclusion of the N-heteroatom in the graphitic structure increases the carbon’s conductivity and electron transport in the conduction band [19]. Various biomass, i.e. apricot shell [20], waste tea bag [21], chitosan [22], glucose [23], lignin [24] as carbon sources produced by HTC method have been studied as electrode in organic electrolytes Na-ion batteries or supercapacitor applications.

Moving from organic electrolyte to the use of aqueous electrolyte, it is a straightforward method to avoid the high cost and safety problems associated with organic liquid electrolytes. The abundance of water, utilizing inexpensive sodium salts such as Na_2_SO_4_, NaNO_3_, NaCl whose ionic conductivity is about 10 times higher than that of organic electrolytes make these electrolytes even more attractive. High ionic conductivity enables to obtain much more cycles capacity. However, the major disadvantage of aqueous electrolyte batteries is the low thermodynamic stability of water (1.23 V) that results in low energy density [2, 25–27]. Gogotsi and Dyatkin reported the specific capacitance of the porous carbon spheres in which 138 F/g at 2 mV/s and 91 F/g at 100 mV/s in 1.0 M Na_2_SO_4_ aqueous electrolyte were obtained [28]. Whitacre et al. demonstrated that specific capacitance values of 200 F/g in an aqueous Na_2_SO_4_ electrolyte can be achieved with hard carbons synthesized from low cost food-grade carbohydrates [2]. Sevilla et al. found that the supercapacitor performances of N-doped carbon from glucosamine/carbon nanotube composites were 50~60 F/g in 1.0 M H_2_SO_4_ electrolyte [29]. Lu et al. prepared coin type supercapacitor electrodes from high surface area activated carbon produced from corn by hydrothermal method. The capacitance values in aqueous, organic and inorganic electrolytes reached to 222 F/g, 202 F/g and 188 F/g, respectively [30]. Altinci and Demir synthesized a sponge-like porous carbon with a high surface area by using the hydrothermal carbonization (HTC) method of pistachio shells with the activation step. They reported 166 F/g in capacitance value in 1 M KOH electrolyte at 0.5 A/g current density [31].

The aim of this study is to accomplish high surface area, porous, amorphous N-doped carbons from glucosamine precursor with HTC method that allow the adsorption-desorption of sodium ions and to investigate their electrochemical performance. For this purpose, carbonaceous material derived from glucosamine were carbonized in inert nitrogen environment at different temperatures (500, 750, 1000 °C). By utilizing scanning electron microscopy (SEM), X-Ray diffraction (XRD), Fourier transform infrared spectroscopy (FT-IR), thermogravimetric analysis (TGA) and Brunauer-Emmett-Teller (B.E.T), morphological and structural examinations were investigated. Electrochemical measurements were performed by cyclic voltammetry (CV), galvanostatic charge/discharge test and electrochemical impedance spectroscopy (EIS).

## 2. Materials and methods

### 2.1. Synthesis of glucosamine derived n-doped carbon

Commercially available 2.0 g of D(+)-glucosamine.HCl was mixed in 18 g distilled water in a magnetic stirrer for 45 min. Then the aqueous solution of D(+)-glucosamine.HCl was placed in a Teflon inlet autoclave and kept for 20 hours in a furnace at 180 °C to proceed hydrothermal carbonization (HTC) process. According to the HTC synthesis route, the applied temperature (180 °C) is sufficient enough to first dehydration of glucosamine and then complete dehydration to form carbonaceous materials [17,19,29] After HTC step, the carbonaceous material was washed with water and ethanol by soxhlet extraction then placed in a vacuum furnace overnight to dry. The resulting sample was quoted as GA-HTC. Afterwards, different carbonization temperatures 500, 750, 1000 °C were applied in a tubular furnace with an inert N_2_ gas for 6 hours in order to improve the conductivity. Further carbonized N-doped carbons derived from glucosamine were named as GA-500, GA-750 and GA-1000, respectively.

### 2.2. Material characterization

Morphological characterizations of the synthesized carbons were investigated by scanning electron microscope (SEM Philips XL30). Thermogravimetric analyses, TGA, were performed using Perkin Elmer 4000 instrument at the temperature between 30–700 °C with a 10 min/°C heating range in an inert nitrogen gas environment. X-ray diffraction (Bruker D8 diffractometer 2θ mode, Cu Kα radiation, λ = 1.5406 nm) patterns of the sample were recorded in the range of 2θ = 0–90°. Surface area and pore size distribution evaluation of hydrothermal carbonization carbon sample and carbonized N-doped carbon samples were determined by using the multi point Brunauer-Emmett-Teller (B.E.T) analyzer (Quantachrome Autosorb Instruments) via nitrogen adsorption isotherms at 77 K under vacuum. FTIR spectroscopy (Perkin Elmer Spectrum 100) was used to determine the bonds of N-doped carbon samples (GA-500, GA-750, GA-1000) and bare hydrothermal carbon, GA-HTC.

### 2.3. Electrochemical measurements

For the electrochemical tests, initially electrodes were prepared by a slurry formation in which 80 wt.% active mass of the N-doped carbon derived from glucosamine (GA-500, GA-750, GA-1000), 10 wt.% of Ketjen black (KB) conductive additive and 10 wt.% of polyvinylidene fluoride (PVDF) binder were mixed in N-methyl-2-pyrrolidone (NMP) solution for 20 h. Later, electrode slurry was coated on the graphite plate in the form of a thin film to have an area of 2 cm × 1 cm. The working electrode has ~2.0 mg active material loading and ~30 μm coating thickness. For the counter electrode, a cleaned graphite plate with a blank surface was used. Half-cell test measurements were carried out in two operating voltage range (0.0 – 0.8) V and (between −0.8 and 0.0) V according to the 3-electrode cell configuration. Ag/AgCl electrode was used as the reference electrode and 1.0 M Na_2_SO_4_ dissolved in bi-distilled water was used as aqueous electrolyte. Electrochemical Impedance Spectroscopy (EIS) was carried out between 0.1 mHz and 0.2 MHz with a magnitude of 5 mV voltage.

## 3. Results and discussion

### 3.1. Synthesis and characterization

**Figure 1:**
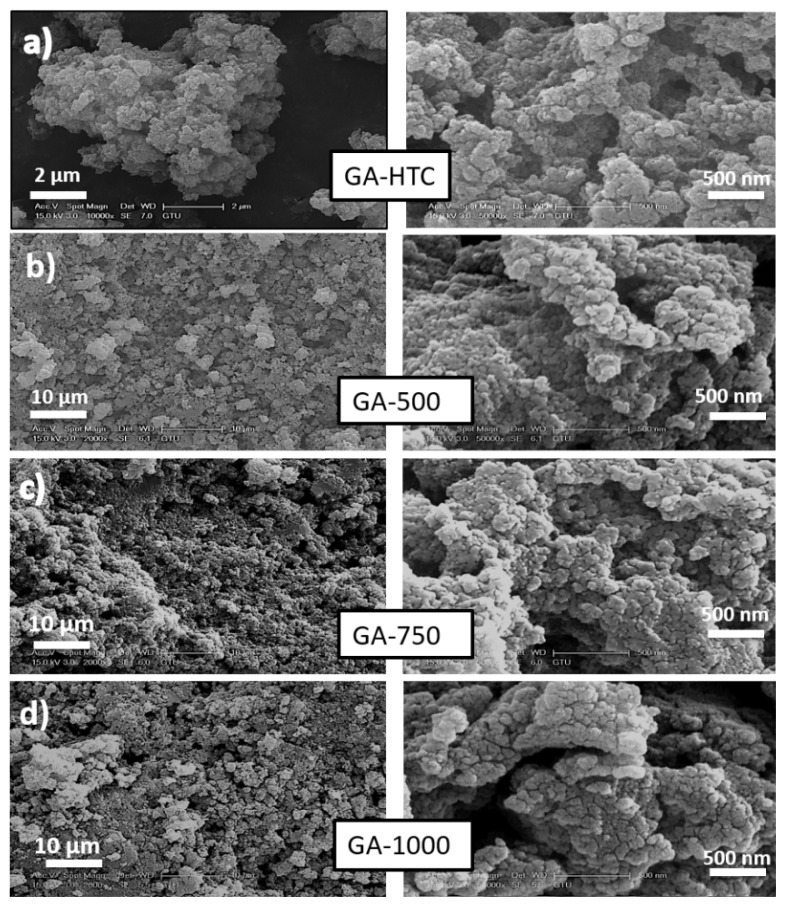
SEM images of a) GA-HTC, b) GA-500, c) GA-750, and d) GA-1000 at different display sizes.

Morphological investigations of carbonaceous materials which was synthesized from glucosamine sources via hydrothermal carbonization (HTC) method was firstly determined by SEM in Figure 1. HTC is a simple, safe and inexpensive method to obtain carbonaceous structures at low temperature, using only water and pure glucosamine precursors as an input. On the other hand, the resulting particles have full of functional groups and low electronic conductivity in nature. Thus, at varying temperatures (500, 750, 1000 °C) the samples were further heat treated in an inert nitrogen gas environment. The intercalation of the nitrogen heteroatom into the graphitic structure with high temperature has been contributed to the electron transport in the conduction band [32]. From the SEM images, agglomerated and porous texture can be clearly observed independently of the type of the samples. Even though the changes in particle size are not visible upon heat treatment, the structure turned out to be more porous that is beneficial for Na-ion adsorption/desorption during the electrochemical cell performances.

**Figure 2:**
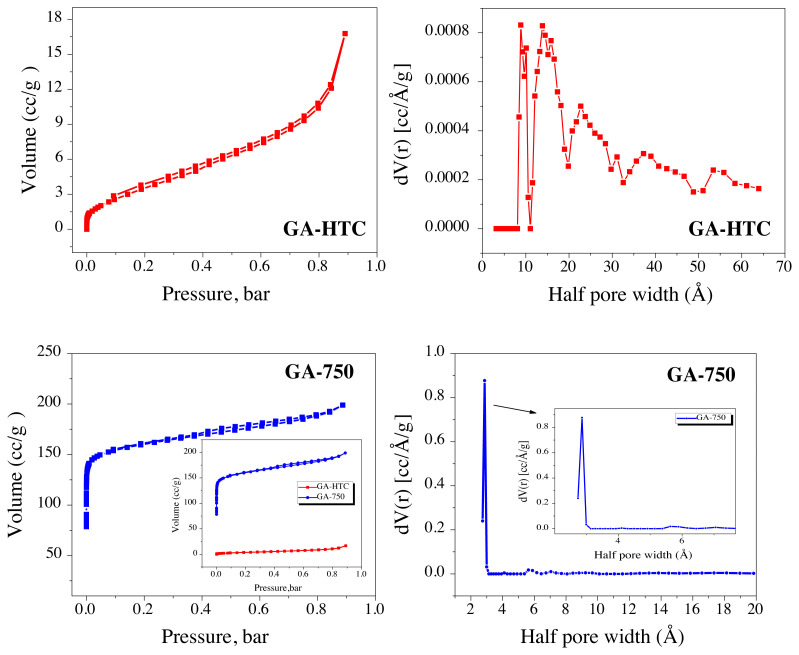
a) N_2_ adsorption-desorption isotherm at 77K of GA-HTC, b) pore size distribution of GA-HTC, c) N_2_ adsorption-desorption isotherm at 77K of GA-750, d) pore size distribution of GA-750.

The surface area of all the synthesized carbon materials was determined by nitrogen adsorption-desorption isotherm at 77K by Brunauer-Emmett-Teller (BET) analysis. Isotherm and pore width graphs shown in Figures 2a–2d). There is a clear change at the isotherm curves and pore size distribution of the materials depending on the temperature treatment. While micro and mesopores were distributed in the hydrothermal carbonized glucosamine sample GA-HTC, the presence of upward micropores with a sharp distribution was observed in the GA-750 sample, which was carbonized at 750 °C. Therefore, a high surface area has been obtained at high temperature carbonization as expected. In other words, it can be said that the material has been carbonized at high temperatures and a more porous structure was provided. Adsorption isotherms type (IV-V) hysteresis are shown for a mesoporous and microporous substance according to the IUPAC isotherm classification. Figure 2 desorption hysteresis can be often connected with narrow pores [33]. B.E.T method to derive the surface area from adsorption-desorption isotherm data, thus, Equation (1) was used for the B.E.T linear isotherm equation below:


(1) $$\frac{P}{V\times (P_{0}-P)}=\frac{1}{Vm\times C}+\frac{\left(C-1\right)}{Vm\times C}\times \left(\frac{P}{P_{0}}\right)$$

Where the (P/P_0_) term is relative pressure, C is the B.E.T constant that is the intercept at the linearization fit, V_m_ is the monolayer adsorbed gas quantity (cc/g) [34].

BET surface areas of the GA-HTC and GA-750 carbon samples are 14.047 and 610.368 cm^2^/g and the total pore volumes are 0.018 cc/g and 0.278 cc/g, respectively. It is clearly seen in Figure SI-1a and Figure SI-1b, the surface area of the GA-750 sample increased considerably after the carbonization process in the nitrogen environment after HTC. It can be said that the carbonization treatment effectively increases the surface area and total pore volume of the carbon sample. When looking at the pore size distribution graphs that determined by the density functional theory (DFT), the GA-HTC sample has micro and mesoporous structure although the GA-750 sample mostly has micropores of approximately 0.3 nm in size that stated in the subgraph (Figure 2d). The carbon structures having micropores and mesopores facilitate the adsorption and desorption of sodium ion species into the structure and increases the diffusion rate by shortening the diffusion pathway. Adsorption-desorption isotherms were summarized at Table 1.

**Figure 3:**
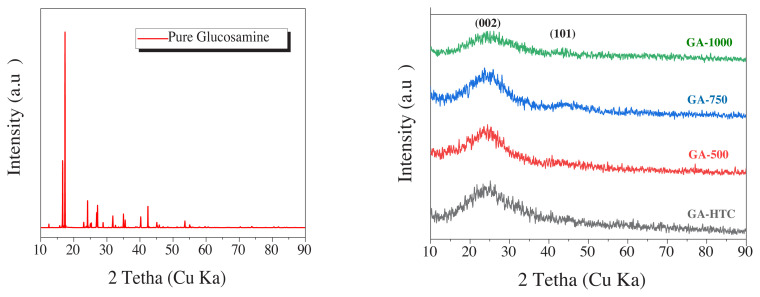
XRD patterns of the a) glucosamine precursor, b) GA-HTC, GA-500, GA-750, GA-1000.

As seen in Figures 3a–3b), bare glucosamine diffraction pattern showed a crystalline structure and had a sharp characteristic peaks. Conversely, an amorphous carbon typical wide peak was observed at 23° (002) peak plane position for hydrothermal carbonization glucosamine (GA-HTC) and other samples carbonized at different temperatures (GA-500, GA-750 and GA-1000). As the carbonization temperature increases, the weak (101) graphitic carbon layer peak appears around 43° that is ascribed to regular turbostractic carbon structure as well as increase of the amount of nitrogen [20,22,35]. Scherrer and Bragg equation were used for the calculation of the crystalline plane size and graphitic carbon interlayer space. d (002) and crystallite sizes of carbon samples were determined and summarized in Table 2. The interlayer distance of the carbonaceous glucosamine via hydrothermal carbonization at 180 °C was found to be approximately 0.7 nm, and, as the temperature increased, distance narrowed to 0.58 – 0.56 nm. Since this distance is wider than the distance between graphene layers, it created a favorable distance for the insertion of sodium ion.

**Figure 4:**
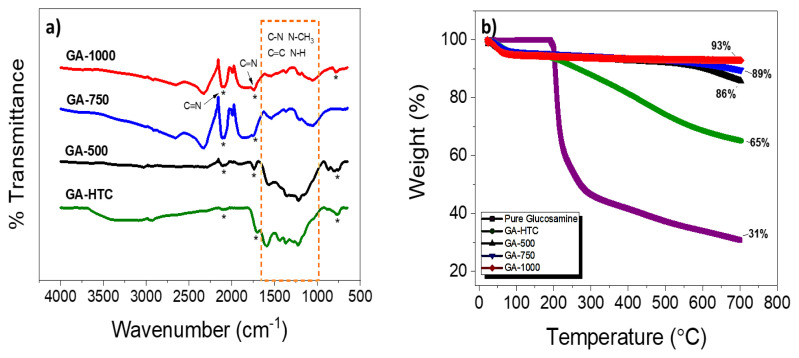
a) FTIR spectroscopy of hydrothermal carbonization samples treated at different temperatures, b) thermogravimetric analyses (TGA) curves under the inert nitrogen atmosphere between 10–700 °C.

In the FTIR analysis of N-doped carbons inherited from glucosamine, the peak at 700 cm^−1^ indicated by a pointed star was attributed to the bending mode in graphite-like areas with nitrogen atoms [36]. The presence of N atoms in the carbon network was evident by the C - N and N - CH_3_ bonds at 1250 – 1372 cm^−1^ and 1200 – 1600 cm^−1^. C = N and C - O in amides showing repetitive units in glucosamine appearing at nearly 1650 cm^−1^ – 1590 cm^−1^ were attributed to stretch vibration. The peaks observed between 1450 and 1250 cm^−1^ correspond to the bond groups of C = C, C = N and C = CO, respectively. The band at 1621 cm^−1^ can be associated with different groups particularly the C = N stretch vibration or the C - O stretch vibration in amides [37]. The band at 1252 cm^−1^ can be related to C - O stretching vibration, C - C skeleton, C - N and N - H stretch and bending in amides [38]. All these peaks referred as dash line in FTIR spectra (Figure 4a). The sharp peak appearing between 2100 – 2300 cm^−1^ for the GA-750 and GA-1000 carbons can be associated with the C ≡ N band [37].

When looking at the thermogravimetric analysis curves with an inert nitrogen gas in the range of 10–700 °C temperature in Figure 4b, the pure glucosamine has lost approximately 69% of its weight. On the other hand, GA-1000, GA-750, and GA-500 have only small weight loss resulting from the unbound water at approximately 100 °C in which they kept their remaining mass around 93 wt.%, 89 wt.%, and 86 wt.%, respectively. After 100 °C, high amount of carbons was obtained by GA-1000, GA-750 and GA-500. However, GA-HTC sample lost its unbound water similarly then degraded and lost about 35 wt% indicating that GA-HTC has full of functional groups as depicted at the FTIR in Figure 4a.

### 3.2. Electrochemical measurements

Cyclic voltammetry (CV) and galvanostatic charge/discharge electrochemical measurements were performed in 1.0 M Na_2_SO_4_ (pH ≈ 5.8–6.0) solution that offers safe, cheap and effective technology in comparison with organic electrolytes. Half-cell tests were run in a 3-electrode configuration lab-scale system using two types of voltage ranges i) (between −0.8 and 0.0) V negative voltage and ii) (0.0–0.8) V positive voltage at 0.37 A/g current density against to Ag/AgCl reference electrode.

The absence of any reduction-oxidation peaks on the CVs in the aqueous electrolyte solution demonstrate that N-doped hard carbon acts as a non-Faradic capacitive electrode, and ions diffuse in the structure with the principle of adsorption and desorption on the amorphous surface [39]. Since the oxidation and reduction reaction did not occur for the carbon anode in aqueous electrolyte half-cell experiments, charge-discharge capacitance were expressed as farad per gram active substance in capacity calculations.

**Figure 5:**
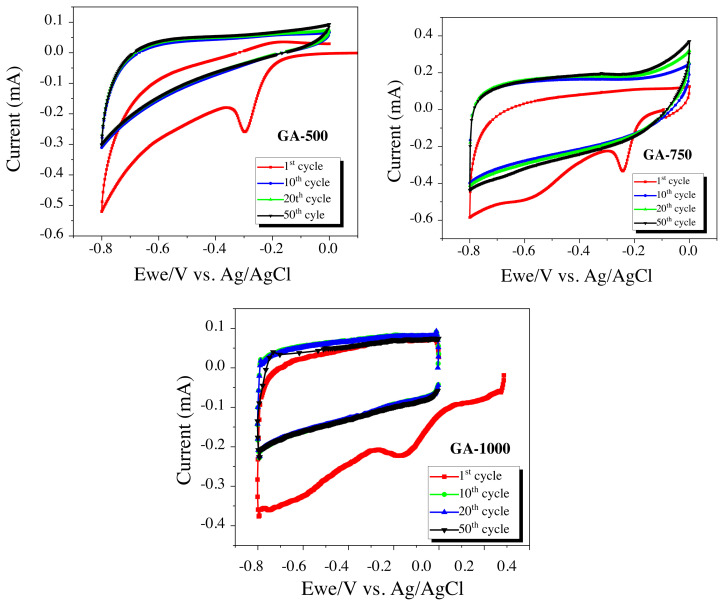
Cyclic voltammogram at 1 mV/s scan rate in 1.0 M Na_2_SO_4_ electrolyte, a) GA-500, b) GA-750 c) GA-1000.

**Figure 6:**
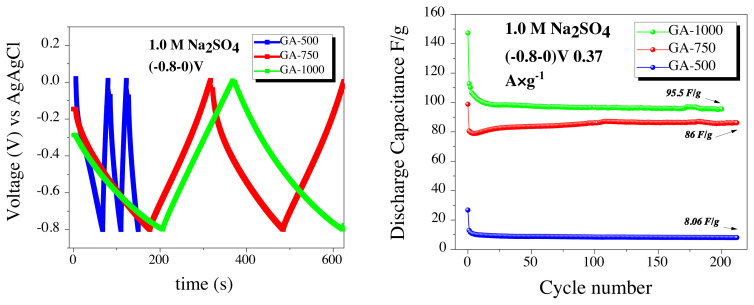
Comparison of a) Galvanostatic voltage vs time graphs, b) capacitance performances of GA-500, GA-750, and GA-1000 carbons at (between −0.8 and 0.0) V.

GA-500, GA-750 and GA-1000 cyclic voltammetry, galvanostatic measurements at (between −0.8 and 0.0) V voltage window were given in Figure 5 and Figure 6, respectively. The peak seen in the first cycle around (–0.3) V in Figure 5 may result from the presence of functional groups that have not been completely reduced during the carbonization step. Since same CV peaks existed in the first cycles appeared at the other carbon samples, it could be said that the nonreduced functional structures remain in the carbonization step. In later cycles, typical rectangular shaped curves have been obtained in CV curves of carbon electrodes meaning that no reduction/oxidation peak in the voltammogram.

Considering the galvanostatic cycling shown in Figure 6, all three electrodes performed a characteristic capacitive behavior with a triangle voltage-time curves. GA-500 reached a very low capacitance values of 8.06 F/g at 0.37 A/g in (between −0.8 and 0.0) V negative voltage range, which cannot be compared with other GA-750 and GA-1000 carbons. The GA-750 sample, has a stable discharge capacity while its capacity has reached an acceptable result in 1.0 M aqueous electrolyte recorded as 86 F/g at the (between −0.8 and 0.0) V. Lastly, galvanostatic capacity measurement of the GA-1000 sample lead the value of 95.5 F/g. Sum of the negative voltage performances of GA-500, GA-750 and GA-1000 in 1.0 M Na_2_SO_4_ at Figure 6, the samples showed stable cycle performances over near 200 cycles, and the best discharge capacitance attained for the GA-1000.

**Figure 7:**
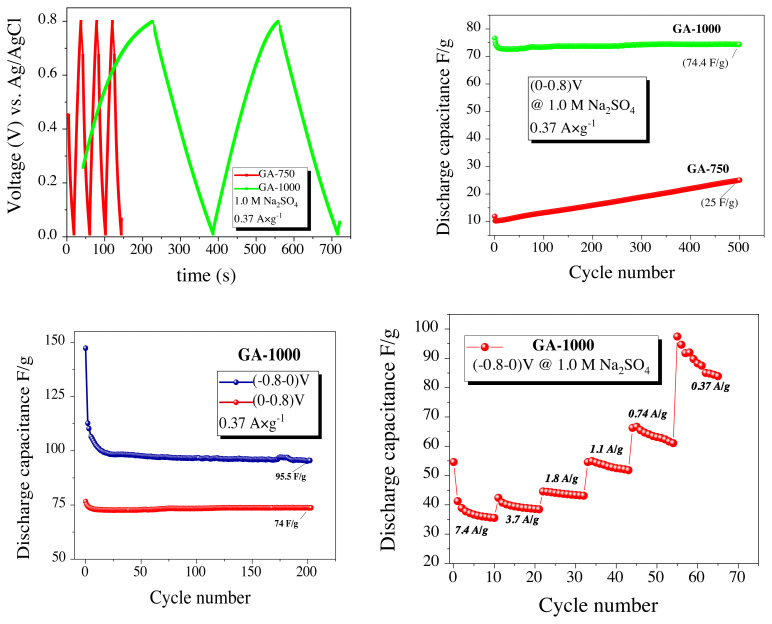
Comparison of a) galvanostatic voltage vs time, b) capacitance performances of GA-750 and GA-1000 at (0.0–0.8) V, c) the capacity performance of GA-1000 between (−0.8–0.0) and (0.0–0.8) V voltage windows, d) various current density (A/g) capability performances of GA-1000 at (−0.8–0.0) V.

As a second set of experiments, the measurements performed in parallel with the positive voltage (0.0–0.8) V operating range however almost no capacity was obtained from the GA-500 electrode. Capacitance values for GA-750 and GA-1000 realized in 1.0 M electrolyte concentrations are shown in Figures 7a–7d). N-doped carbon samples resulted sufficient performances at the negative voltage, whereas they could not reach to high performances while working at positive voltage. The reason could be explained by the surface charges of those carbon that have positive zeta-potential after the first hydrothermal carbonization step. As depicted in the literature, the zeta potentials shift to negative region when the carbonization temperatures increase from 750 – 1000 °C, thus the surface of the electrodes are negatively charged at the working pH value [40]. Therefore, as proven in Figure 7c, the best performance was found at the negative operating voltage rather than at the positive voltages.

C-rate capacities of GA-1000 as an anode electrode were tested at (between −0.8 and 0.0) V in the negative voltage range for 10 cycles from high current density (7.4 A/g) to low current density (0.37 A/g) (Figure 7c). When discharged at a current density of 7.4 A/g, the capacity value was approximately 35 F/g. When the current density was reduced to 0.37 A/g, the highest discharge capacity was obtained as shown in Figure 7d.

The electrochemical impedance spectrum of GA-1000 was inquired in order to reveal the sodium ion diffusion property of glucosamine derived N-doped porous carbon electrode. The Warburg impedance on the Nyquist plot gives a straight linear part with a 45 ° phase in the EIS. 45 ° line at the low frequency region on the Nyquist chart can be associated with sodium-ion diffusion. In energy storage systems, the porosity of the electrodes causes a similar characteristic 45° line on the Nyquist chart [41]. The charge transfer resistance at the solid electrolyte interface is attributed to a semicircle at high frequency. From the semicircular endpoint data from the high frequency region to the low frequency region, the total resistance of the electrochemical test cell can be estimated [42,43]. Sodium ion diffusion coefficient was estimated from Equation (2) via the Warburg low frequency diffusion estimation.


(2) $$D_{\left(Na^{+}\right)}=\frac{R^{2}\times T^{2}}{\left(2\times A^{2}\times n^{4}\times F^{4}\times C^{2}\times \sigma^{2}\right)}$$

**Figure 8:**
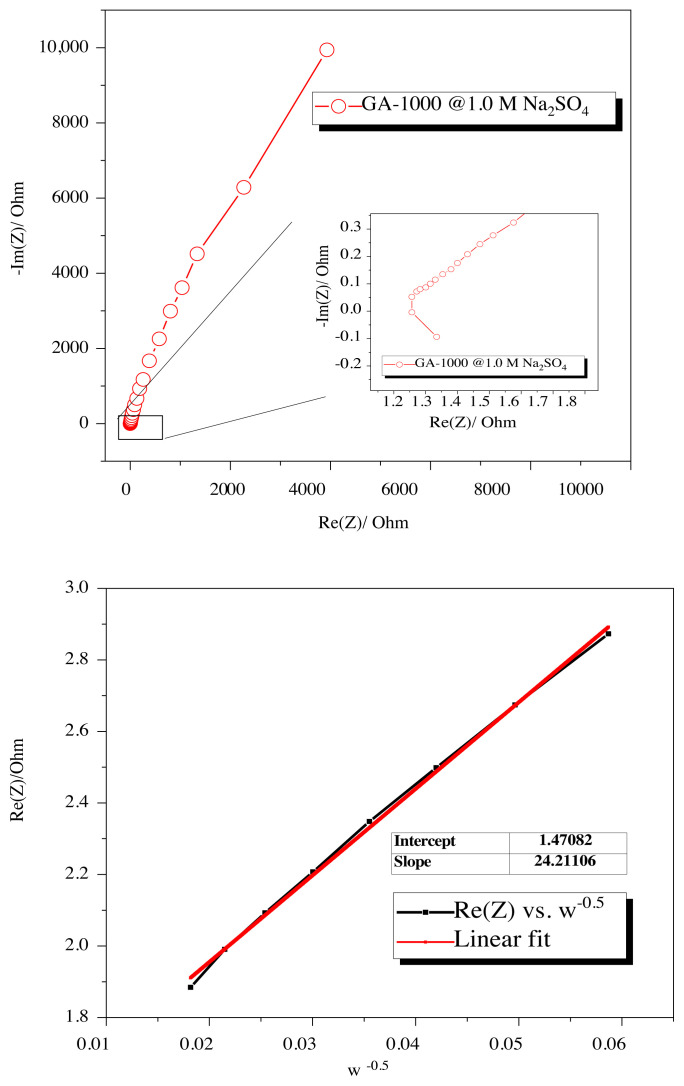
a) Electrochemical impedance spectrum of GA-1000 b) Relationship with real impedance (Z_Re_) and angular frequency (ω^−0.5^).

Where R is the absolute gas constant, T is the room temperature (298 K), A is the electrode surface area (2 × 1 cm^2^), n is the number of electron transferred, F is the Faraday constant (96,500 C/mol), C is concentration of Na^+^ (1.0 M) and σ is the Warburg factor, which was calculated from the slope of real impedance (Z_Re_) versus the angular frequency (ω^−0.5^) shown in Figure 8–8b) with using the Equation (3) below.


(3) $$Z_{Re}=R_{s}+R_{ct}+\sigma \omega^{-0.5}$$

Sodium ion diffusion coefficient of GA-1000 was found to be 1.5 × 10^−14^ cm^2^/second. Xiao et al. obtained hard carbon nanoparticles by the pyrolysis of polyaniline which resulted around 10^−13^–10^−15^ cm^2^/second sodium ion diffusion coefficient using Warburg impedance approach [44]. In addition, Na^+^ diffusivity was found in the rage of 10^−12^ – 10^−15^ cm^2^/second of the kelp-derived hard carbon electrodes [45]. Thus, according to the literature reports, the resulting GA-1000 anode has a reasonable diffusion coefficient value.

## 4. Conclusion

In this study, N-doped amorphous carbons were synthesized derived from D(+)-glucosamine.HCl as a source via the inexpensive, safe, one-step hydrothermal carbonization method. Subsequently, hydrothermal carbon (GA-HTC) was carbonized at 500, 750 and 1000 °C under N_2_ gases atmosphere in order to increase the surface area and electrical conductivity of the resulting electrodes. The characterization of the synthetized amorphous carbon materials was made using SEM, XRD, FTIR, TGA, and B.E.T adsorption-desorption isotherm. These characterizations supported the successful synthesis of the amorphous N-doped carbons with high surface facilitating easy adsorption-desorption of the Na-ion species into the carbon structure. These N-doped carbons performed properly when used as electrodes in energy storage systems owing to microporosity and the presence of nitrogen heteroatoms functionalities in the structure. Electrochemical data have collected using cyclic voltammetry and galvanostatic charge/discharge method in 1.0 M Na_2_SO_4_ aqueous electrolyte at two different voltage ranges (between −0.8 and 0.0) V and (0.0–0.8) V. These parameters indicated that non-Faradic capacities depend on the operating voltage range and carbonization temperatures. The capacitance values were found to be 8.06 F/g, 86.9 F/g and 95.5 F/g for GA-500, GA-750 and GA-1000, respectively, at negative voltage. On the other hand, much lower values were obtained at the positive voltage (0.0–0.8 V) due to the negatively charged surfaces of the electrodes. EIS measurements were applied to the highest performed electrode (GA-1000) and its Na ion diffusion coefficient was calculated to be 1.5 × 10^−14^ cm^2^/s that is comparable with the literature values.

## Supporting information

Figure SI-1 a) Multipoint BET linear isotherm of GA-HTC, b) Multipoint BET linear isotherm of GA-750 according to Eq (1).

## Figures and Tables

**Table 1:** Adsorption-desorption properties of N-doped carbons driven from glucosamine.

Samples	S_BET_ (m^2^/g)	V_Total_ (cc/g)	V_mic_ (cc/g)	V_mic_/V_Total_	V_meso_/V_Total_	Half pore radius (Å)
GA-HTC	14.047	0.018	0.005	0.28	0.72	8.86
GA-750	610.368	0.278	0.207	0.74	0.26	2.87

**Table 2:** XRD data of the pure glucosamine and carbons derived from glucosamine that carbonized at different temperatures.

Samples	L (cystalline plane, nm)	d_002_ (interlayer space, nm)
GA-HTC	0.344	0.727
GA-500	0.495	0.589
GA-750	0.606	0.581
GA-1000	0.606	0.566
Pure glucosamine	71.433	0.617

## References

[1] Ellis BL Nazar LF Sodium and sodium-ion energy storage batteries Current Opinion in Solid State and Materials Science 2012 16 4 168 177 10.1016/j.cossms.2012.04.002

[2] Whitacre JF Wiley T Shanbhag S Wenzhuo Y Mohamed A An aqueous electrolyte, sodium ion functional, large format energy storage device for stationary applications Journal of Power Sources 2012 213 255 264 10.1016/j.jpowsour.2012.04.018

[3] Herrington R Mining our green future Nature Reviews Materials 2021 6 456 458 10.1038/s41578-021-00325-9

[4] Vaalma C Buchholz D Weil M Passerini S A cost and resource analysis of sodium-ion batteries Nature Reviews Materials 2018 3 1 11 10.1038/natrevmats.2018.13

[5] Greim P Solomon AA Breyer C Assessment of lithium criticality in the global energy transition and addressing policy gaps in transportation Nature Communications 2020 11 1 1 11 10.1038/s41467-020-18402-y PMC7486911 32917866

[6] Tarascon J Is lithium the new gold? Nature Publishing Group 2010 2 6 510 10.1038/nchem.680 20489722

[7] Armand M Tarascon JM Building better batteries Nature 2008 451 7179 652 657 10.1038/451652a 18256660

[8] Tarascon JM Na-ion versus Li-ion Batteries: Complementarity Rather than Competitiveness Joule 2020 4 8 1616 1620 10.1016/j.joule.2020.06.003

[9] Zhang L Zhao XS Carbon-based materials as supercapacitor electrodes Chemical Society Reviews 2009 38 9 2520 2531 10.1039/b813846j 19690733

[10] Kim SW Seo DH Ma X Ceder G Kang K Electrode materials for rechargeable sodium-ion batteries: Potential alternatives to current lithium-ion batteries Advanced Energy Material 2012 2 7 710 721 10.1002/aenm.201200026

[11] Simon P Gogotsi Y Materials for electrochemical capacitors Nature Materials 2008 7 11 845 854 10.1038/nmat2297 18956000

[12] Huang Y Zheng Y Li X Adams F Luo W Electrode materials of sodium-ion batteries toward practical application American Chemical Society Energy Letters 2018 3 7 1604 1612 10.1021/acsenergylett.8b00609

[13] Titirici MM Antonietti M Chemistry and materials options of sustainable carbon materials made by hydrothermal carbonization Chemical Society Reviews 2010 39 1 103 116 10.1039/b819318p 20023841

[14] Demir-Cakan R Baccile N Antonietti M Titirici MM Carboxylate-rich carbonaceous materials via one-step hydrothermal carbonization of glucose in the presence of acrylic acid Chemistry of Materials 2009 21 3 484 490 10.1021/cm802141h

[15] Yu L Falco C Weber J White RJ Howe JY Carbohydrate-derived hydrothermal carbons: A thorough characterization study Langmuir 2012 28 33 12373 12383 10.1021/la3024277 22853745

[16] Titirici MM Antonietti M Chemistry and materials options of sustainable carbon materials made by hydrothermal carbonization Chemical Society Reviews 2010 39 1 103 116 10.1039/b819318p 20023841

[17] Cakan RD Synthesis, characterization and applications of nanostructured materials using hydrothermal carbonization PhD Max Planck Institute for Colloid and Interfaces Germany 2009

[18] Sevilla M Fuertes AB The production of carbon materials by hydrothermal carbonization of cellulose Carbon 2009 47 9 2281 2289 10.1016/j.carbon.2009.04.026

[19] Zhao L Baccile N Gross S Zhang Y Wei W Sustainable nitrogen-doped carbonaceous materials from biomass derivatives Carbon 2010 48 13 3778 3787 10.1016/j.carbon.2010.06.040

[20] Demir E Aydin M Arie AA Demir-Cakan R Apricot shell derived hard carbons and their tin oxide composites as anode materials for sodium-ion batteries Journal of Alloys and Compounds 2019 788 1093 1102 10.1016/j.jallcom.2019.02.264

[21] Arie AA Tekin B Demir E Demir-Cakan R Hard carbons derived from waste tea bag powder as anodes for sodium ion battery Materials Technology 2019 34 9 515 524 10.1080/10667857.2019.1586087

[22] Aydin M Demir E Unal B Dursun B Ahsen AS Chitosan derived N-doped carbon coated SnO2 nanocomposite anodes for Na-ion batteries Solid State Ionics 2019 341 3 115035 10.1016/j.ssi.2019.115035

[23] Titirici MM Alptekin H Au H Jensen ACS Olsson E Sodium storage mechanism investigations through structural changes in hard carbons ACS Applied Energy Materials 2020 3 10 9918 9927 10.1021/acsaem.0c01614

[24] Demir M Tessema TD Farghaly AA Nyankson E Saraswat SK Lignin-derived heteroatom-doped porous carbons for supercapacitor and CO2 capture applications International Journal of Energy Research 2018 42 8 2686 2700 10.1002/er.4058

[25] Demir-Cakan R Palacin MR Croguennec L Rechargeable aqueous electrolyte batteries: From univalent to multivalent cation chemistry Journal of Materials Chemistry A 2019 7 36 20519 20539 10.1039/c9ta04735b

[26] Bin D Wang F Tamirat AG Suo L Wang Y Progress in aqueous rechargeable sodium-ıon batteries Advanced Energy Materials 2018 8 17 1 31 10.1002/aenm.201703008

[27] Kim H Hong J Park KY Kim H Kim SW Kang K Aqueous rechargeable Li and Na ion batteries Chemical Reviews 2014 114 23 11788 11827 10.1021/cr500232y 25211308

[28] Zhang C Hatzell KB Boota M Dyatkin B Beidaghi M Highly porous carbon spheres for electrochemical capacitors and capacitive flowable suspension electrodes Carbon 2014 77 155 164 10.1016/j.carbon.2014.05.017

[29] Sevilla M Yu L Zhao L Ania CO Titirici MM Surface modification of CNTs with N-doped carbon: An effective way of enhancing their performance in supercapacitors ACS Sustainable Chemistry and Engineering 2014 2 4 1049 1055 10.1021/sc500069h

[30] Lu Y Zhang S Yin J Bai C Zhang J Mesoporous activated carbon materials with ultrahigh mesopore volume and effective specific surface area for high performance supercapacitors Carbon 2017 124 64 71 10.1016/j.carbon.2017.08.044 PMC5997976 29904649

[31] Altinci OC Demir M Beyond conventional activating methods, a green approach for the synthesis of biocarbon and ıts supercapacitor electrode performance Energy and Fuels 2020 34 6 7658 7665 10.1021/acs.energyfuels.0c01103

[32] White RJ Antonietti M Titirici MM Naturally inspired nitrogen doped porous carbon Journal of Materials Chemistry 2009 19 45 8645 8650 10.1039/b911528e

[33] Alothman ZA A review: Fundamental aspects of silicate mesoporous materials Materials 2012 5 12 2874 2902 10.3390/ma5122874

[34] Ada K Sarıkaya Y Alemdaroğlu T Onal M The investigation of the pore structure of fine alumina powders prepared for ceramic production by the technique of precipitation from solution Communications Faculty of Sciences University of Ankara Series B 2000 46 57 67

[35] Baccile N Antonietti M Titirici MM One-step hydrothermal synthesis of nitrogen-doped nanocarbons: Albumine directing the carbonization of glucose ChemSusChem 2010 3 2 246 253 10.1002/cssc.200900124 19885901

[36] Sung SL Tseng CH Chiang FK Guo XJ Liu XW Shih HC Novel approach to the formation of amorphous carbon nitride film on silicon by ECR-CVD Thin Solid Films 1999 340 1 169 174 10.1016/S0040-6090(98)01465-5

[37] Fay DL Structure and Determination of Organic Compounds: Tables of Spectral Data Angewandte Chemie International Edition Berlin Heidelberg Springer 2009

[38] Laginhas C Nabais JMV Titirici MM Activated carbons with high nitrogen content by a combination of hydrothermal carbonization with activation Microporous and Mesoporous Materials 2016 226 125 132 10.1016/j.micromeso.2015.12.047

[39] Zhang L Zhao XS Carbon-based materials as supercapacitor electrodes Chemical Social Review 2009 38 7 2520 2531 10.1039/B813846J 19690733

[40] Zhao L Baccile N Gross S Zhang Y Wei W Sustainable nitrogen-doped carbonaceous materials from biomass derivatives Carbon 2010 48 13 3778 3787 10.1016/j.carbon.2010.06.040

[41] Huang J Diffusion impedance of electroactive materials, electrolytic solutions and porous electrodes: Warburg impedance and beyond Electrochimica Acta 2018 281 170 188 10.1016/j.electacta.2018.05.136

[42] Tang K Fu L White RJ Yu L Titirici MM Hollow carbon nanospheres with superior rate capability for sodium-based batteries Advanced Energy Materials 2012 2 7 873 877 10.1002/aenm.201100691

[43] Dogrusoz M Demir-Cakan R Mechanochemical synthesis of SnS anodes for sodium ion batteries International Journal of Energy Research 2020 44 13 10809 10820 10.1002/er.5735

[44] Xiao L Cao Y Henderson WA Sushko ML Shao Y Hard carbon nanoparticles as high-capacity, high stability anodic materials for Na-ion batteries Nano Energy 2016 19 1 279 288 10.1016/j.nanoen.2015.10.034

[45] Wang P Zhu X Wang Q Xu X Kelp-derived hard carbons as advanced anode materials for sodium-ion batteries Journal of Materials Chemistry A 2017 5 12 5761 5769 10.1039/c7ta00639j

