# Supplementary figures and images for: Glucosamine derived hydrothermal carbon electrodes for aqueous electrolyte energy storage systems
Source: Turk J Chem. 2021 Aug 4;45(6):1678–89. doi: 10.3906/kim-2105-35 (PMC10734747; doi:10.3906/kim-2105-35)

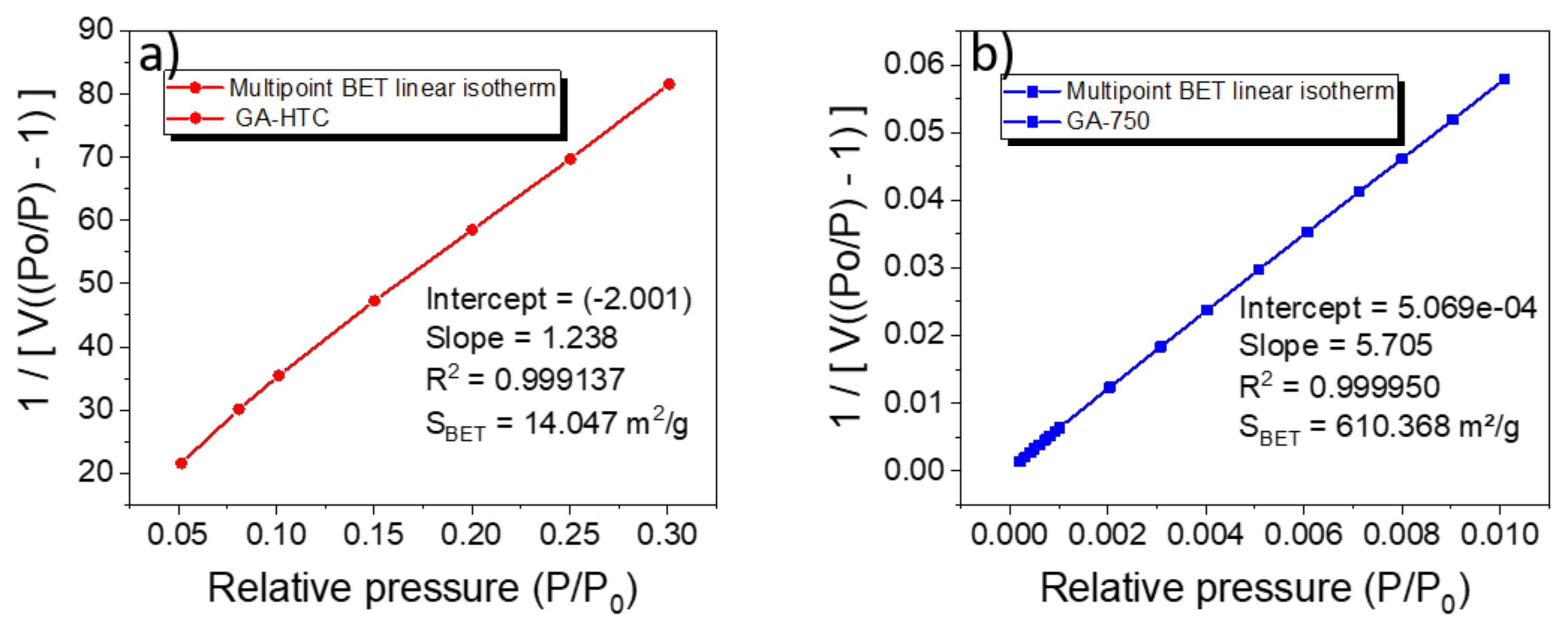

Supplement: Figure SI-1 — a) Multipoint BET linear isotherm of GA-HTC, b) Multipoint BET linear isotherm of GA-750 according to Eq (1). [file turkjchem-45-6-1678s1.tif]
